# Kinesiophobia Post Total Hip Arthroplasty: A Retrospective Study

**DOI:** 10.7759/cureus.15991

**Published:** 2021-06-28

**Authors:** Mohammad K Alsaleem, Abdullah M Alkhars, Hassan A Alalwan, Adia Almutairi, Arwa Alonayzan, Ibrahim A AlYaeesh

**Affiliations:** 1 Orthopaedics, King Fahad Hospital Hofuf, Al-Ahsa, SAU; 2 Orthopedic Surgery, King Abdulaziz Hospital, Al-Ahsa, SAU; 3 Orthopedic Surgery, King Abdulaziz Airbase Armed Forces Hospital, Dhahran, SAU; 4 Orthopedic Surgery, Defence Force hospital, West Riffa, BHR

**Keywords:** kinesiophobia, total hip arthroplasty, rehabilitation, functional outcome of total hip aethroplasty, post operative pain

## Abstract

Introduction

One of the major psychological factors that can affect the outcome of hip arthroplasty is postoperative kinesiophobia, which is defined as a fear of movement. The effect of kinesiophobia and excruciating pain has not been widely explored in hip arthroplasty literature especially in Saudi Arabia.

Aim

This study aimed to investigate kinesiophobia and pain catastrophizing after total hip arthroplasty (THA) in King Fahad Hospital, Hofuf, Saudi Arabia.

Materials and methods

This is a retrospective cross-sectional study conducted at King Fahad Hospital, Hofuf, Saudi Arabia. Using a validated self-administered questionnaire, participants were questioned telephonically. Questionnaires included basic demographic characteristics, Tampa Scale of Kinesiophobia (TSK), and Numerical Analogue Scale (NAS). Data were tabulated in MS Excel and all statistical analyses were performed using Statistical Package for the Social Sciences (SPSS) version 21 (IBM Corp., Armonk, NY).

Results

Seventy-four patients were recruited (60.8% females vs 39.2% males). The TSK mean score was 40.7 (SD 8.88) while the mean NAS score was 5.45 (SD 2.79). The prevalence of kinesiophobia was 62.2%. The statistical test revealed that there was a statistically significant positive correlation between TSK score and NAS score (r=460; p<0.001). Furthermore, kinesiophobia was widely prevalent among patients who had avascular necrosis (p<0.001) and among those who underwent physiotherapy (p=0.044).

Conclusion

There was a high prevalence of kinesiophobia among patients who underwent THA. Pain intensity directly correlated with the presence of kinesophobia.

## Introduction

The hip joint is a ball-and-socket type diarthrodial joint that is known for its stability and ability to bear body weight. It influences the body mechanics and plays a major role in the function of the lower extremity. Total hip arthroplasty (THA) is one of the most successful interventions in orthopedics nowadays and has contributed massively to improving patient’s quality of life, morbidity, and mortality. THA has come a long way since its first implantation in the 1950s, including improvements in surgical technique, technology, and postoperative rehabilitation, which all led to increasing the effectiveness and success rate of hip arthroplasty [1].

The outcomes of hip arthroplasty are highly influenced by patient-specific factors, such as sex, age, body stature, and co-morbidities. It is also influenced by patient psychological factors, which can affect their compliance, rehabilitation, and pain perception [2].

in a study of 850 patients who underwent a total hip replacement, it was reported that depression, poor emotional health, and symptomatic osteoarthritis of another joint played a major role in predicting dissatisfaction one year following arthroplasty [3].

Another major psychological factor that can affect the outcome of hip arthroplasty is post-operative kinesiophobia, which is defined as a fear of movement, and physical activity that had resulted in injury or pain in the past. This is an adaptation of the patients in an acutely painful situation. However, once this becomes a habit, patients tend to get into a vicious cycle of avoidance, that can lead to the development of chronic pain and disability affecting functional outcome and return to the previous level of physical activity [4,5].

The cognitive fear-avoidance model that describes a painful experience is interpreted as threatening or alarming, and it can generate catastrophizing cognitions activity that will result in more pain and re/injury [6]. The patients may have trapped in a cycle of increased fear of pain, more pain, and disability [6]. There are several studies that support the validity of the cognitive fear-avoidance model [7-9].

As far as we found, the effect of kinesiophobia and pain catastrophizing have not been widely explored in hip arthroplasty literature especially in Saudi Arabia. Therefore, we aim to assess kinesiophobia in patients who underwent THA in four to eight weeks post-operatively in the Al-Ahsa region of Saudi Arabia.

## Materials and methods

Study area and data collection

This is a cross-sectional study that was conducted at King Fahad Hospital Hofuf to investigate kinesiophobia and pain catastrophizing after hip arthroplasty and if it is associated with negative functional outcomes.

Following institutional review board approval (20-589E) and IRB registration with OHRP/NIH, USA (IRB00010471), consecutive patients undergoing primary THA for the treatment of avascular necrosis, osteoarthritis, rheumatoid arthritis, and traumatic fracture by a fellowship-trained surgeon were retrospectively enrolled in the study. 

The survey was via telephone, begin with explaining the aim of the study and taking the consent from all the participants before answering any question. Then, the participants were asked to answer seven socio-demographical questions (age, gender, level of education, occupation status, indication of the operation, surgical procedure performed (unilateral/bilateral), and whether physiotherapy was taken).

Kinesiophobia was assessed using the Tampa Scale of Kinesiophobia (TSK) which is a therapist administered scale; consists of 17 questions graded by 4 points ranging from “strongly disagree to strongly agree.” The score varies from 17 to 68 and a score of 37 or above is an indicator of the high level of Kinesiophobia. The intensity of post-operative pain will be assessed using the Numerical Analogue Scale (NAS) for pain where 0 is no pain and 10 is the worst pain imaginable.

All data were collected by medical graduates who received prior training for this task. The time required to complete the questionnaire was 10 minutes.

Patient selection

Seventy-four male and female patients in the age group of 18-75 years who had undergone THA during the study period were included. Patients with any neurological deficit, polytrauma involving the lower extremity, and psychiatric disorders were excluded from this study. No measures of co-morbidity or any specific origin or site of pain were included

Statistical analysis

Descriptive statistics were presented using numbers, percentages, means, and standard deviation, whenever appropriate. The prevalence of kinesiophobia was compared with the baseline characteristics of the patients by using Fischer exact test. P-value <0.05 was considered statistically significant while <0.01 was considered highly statistically significant. A correlation procedure was also conducted to determine the linear agreement between the TSK score and the NAS score. All data analyses were carried out using Statistical Packages for Software Sciences (SPSS) version 21 (Armonk, New York: IBM Corporation).

The prevalence of kinesiophobia was measured using the TSK which is a therapist administered scale consisted of 17 questions graded by 4 points Likert scale ranging from “strongly disagree to strongly agree.” Questions 4, 8, 12, 16 are inversely scored. The score range was from 17 to 68 and a score of 37 or above was considered as kinesiophobia. The intensity of postoperative pain was assessed using the NAS where the participants were asked to mark on a scale of 0-10 where the higher the score the higher the intensity of pain.

## Results

We analyzed 74 patients to measure the prevalence of kinesiophobia among patients who underwent THA. As described in Table 1, the most common age group was 18-40 years old (51.4%) with a similar proportion of females (60.8%) and diplomas or below 60.8% while nearly half of them (48.6%) were unemployed. With respect to their body mass index (BMI), more than two-thirds (35.1%) had normal BMI, 23% were overweight, and 29.7% were obese. Avascular necrosis constitutes the majority of the patients (67.6%). Furthermore, more than a half (51.4%) underwent unilateral hip procedure while 48.6% were bilateral. Of those who underwent bilaterally, approximately 60% reported that the right hip affected more. The proportion of patients who seek physiotherapy treatment was 85.1%. In addition, the mean scores of TSK and NAS were 40.7 and 5.45.

**Table 1:** Baseline characteristics of the patients (n=74)

Study variables	N (%)
Age group	
18–40 years	38 (51.4%)
41–60 years	28 (37.8%)
>60 years	08 (10.8%)
Gender	
Male	29 (39.2%)
Female	45 (60.8%)
Level of education	
Diploma or below	45 (60.8%)
Bachelor or master degree	29 (39.2%)
Occupation	
Employed	29 (39.2%)
Unemployed	36 (48.6%)
Student	09 (12.2%)
BMI level	
Underweight	04 (05.4%)
Normal	26 (35.1%)
Overweight	17 (23.0%)
Obese	22 (29.7%)
Morbidly obese	05 (06.8%)
Indication of operation	
Osteoarthritis	16 (21.6%)
Traumatic fracture	08 (10.8%)
Avascular necrosis	50 (67.6%)
Surgical procedure performed	
Unilateral	38 (51.4%)
Bilateral	36 (48.6%)
If bilateral, which side is effected more?^(n=36)^	
None	07 (19.4%)
Left	08 (22.2%)
Right	21 (58.3%)
Physiotherapy was taken	
Yes	63 (85.1%)
No	11 (14.9%)
	Mean ± SD
TSK score	40.7 ± 8.88
NAS score	5.45 ± 2.79

**Figure 1:**
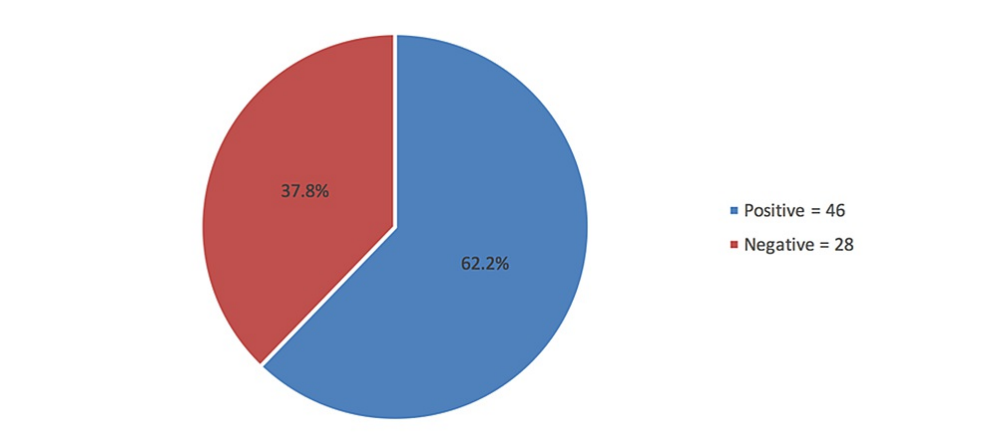
Prevalence of kinesiophobia among patients with total hip arthroplasty

The prevalence of kinesiophobia was shown in Figure 1. It was revealed that nearly two-thirds (62.2%) were classified as having kinesiophobia and the rest were negative (37.8%)

**Figure 2:**
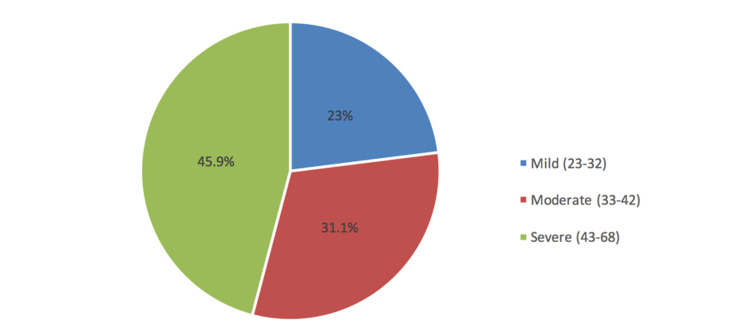
Severity of kinesiophobia

Figure 2 depicted that 45.9% of the patients had severe kinesiophobia, 31.1% were moderate and 23% were mild.

**Figure 3:**
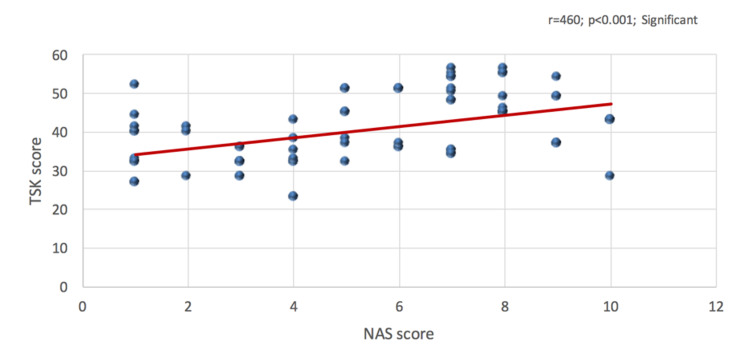
Correlation between Tampa Scale of Kinesiophobia score and Numerical Analogue Scale score

Figure 3 depicted the correlation between TSK and NAS score. It was observed that the correlation between TSK score and NAS score was positively highly statistically significant (r=460; p<0.001), indicating that the increase of kinesiophobia score will also the increase NAS score.

When measuring the relationship between kinesiophobia and the baseline characteristics of the patients, it was found that indication of operation showed a significant relationship with kinesiophobia (p<0.001) where the prevalence of avascular necrosis were statistically significantly higher. Similarly, respondents who seek physiotherapy treatment were statistically significantly more of having kinesiophobia (p=0.044). Other baseline characteristics did not show a significant relationship with kinesiophobia (p>0.05; Table 2).

**Table 2:** Relationship between kinesiophobia and the baseline characteristics of the patients (n=74) *Statistically significant.

Factor	Kinesiophobia		P-value
	Yes N (%) ^(n=46)^	No N (%) ^(n=28)^	
Age group			
18–40 years	24 (52.2%)	14 (50.0%)	1.000
>40 years	22 (47.8%)	14 (50.0%)	
Gender			
Male	17 (37.0%)	12 (42.9%)	0.632
Female	29 (63.0%)	16 (57.1%)	
Level of education			
Diploma or below	28 (60.9%)	17 (60.7%)	1.000
Bachelor or master degree	18 (39.1%)	11 (39.3%)	
Occupation			
Employed	17 (37.0%)	12 (42.9%)	0.944
Unemployed	23 (50.0%)	13 (46.4%)	
Student	06 (13.0%)	03 (10.7%)	
BMI level			
Normal/underweight	20 (43.5%)	10 (35.7%)	0.715
Overweight	11 (23.9%)	06 (21.4%)	
Obese	15 (32.6%)	12 (42.9%)	
Indication of operation			
Osteoarthritis	16 (34.8%)	0	<0.001*
Traumatic fracture	02 (04.3%)	06 (21.4%)	
Avascular necrosis	28 (60.9%)	22 (78.6%)	
Surgical procedure performed			
Unilateral	28 (60.9%)	10 (35.7%)	0.055
Bilateral	18 (39.1%)	18 (64.3%)	
Physiotherapy was taken			
Yes	36 (78.3%)	27 (96.4%)	0.044*
No	10 (21.7%)	01 (03.6%)	

## Discussion

The present study examined 74 patients who underwent THA in either unilateral or bilateral and to determine the effect of kinesiophobia after the intervention. In a postoperative span of four to eight weeks, the prevalence of kinesiophobia was 62.2% (TSK score >37), and 37.8% had a score of ≤37 points which consider them as no kinesiophobia. This result is consistent with the paper of Olsson et al. [10]. The study involved 256 patients (138 controlled group vs 128 intervention group) and the purpose of their investigation was to evaluate person-centered care after hip replacement which was designed to reduce the undesirable impact of low self-efficacy and high level of pain-related fear. Based on their evaluations, more than 50% in both groups (52% control group vs 59% intervention group) had high TSK. They further noted that, of those patients with a high level of TSK, there was a significant shift toward a shorter length of stay most specifically in the intervention group. On the other hand, Manali et al. [11] reported a lower kinesiophobia prevalence rate than our study. Their study involved 52 subjects aged range from 18 to 75 years old. Based on their accounts, 69.2% of the patients had a low level of kinesiophobia which corroborates the study done in Italy [12].

Furthermore, we also noted that the prevalence of kinesiophobia was significantly more on patients who had been diagnosed with avascular necrosis (p<0.001) and those patients who sought physiotherapy treatment (p=0.044). However, the prevalence of kinesophobia were similar across the socio-demographic characteristics of the patients and that includes age, gender, level of education, BMI level, and occupation. These reports are not consistent with the study of Morri et al. [12], based on their accounts, age, sex, and BMI were the significant factors associated with kinesophobia. Similarly, Manali et al. [11] accounted that age was observed to have a positive correlation with TSK score which coincided with the study done in Italy but differed from our study.

Moreover, the findings of this study will contribute to the current evidence that demonstrates kinesiophobia, along with other psychological factors, can prolong or hinder functional improvement in patients who have undergone hip surgery [2,10,12]. In this study, the mean score of NAS was 5.45 (SD 2.79) with a positive highly statistically significant correlation with TSK score (r=460; p<0.001) which generally indicates that the increase in the score of TSK will also increase NAS score. That is since the majority of our subjects had high TSK scores it eventually had a direct effect on the pain score of the patients. This further indicates that patients who were classified as having high kinesiophobia could be an influential factor for the decrease in functional outcome for THA postoperatively which was also validated in this study. This finding is strikingly similar to the paper done in India [10]. Their findings showed that there was a strong positive correlation between pain and the TSK score. However, in a study by Morri et al. [12], they documented that there was no significant correlation between numerical rating scale score and kinesiophobia score postoperatively which were not in accordance with our report.

Limitations of the study

First, strict inclusion and exclusion criteria that were used for the patient selection may have introduced selection bias. Second, no measures of co-morbidity or any specific origin or site of pain were included. Third, multiple factors other than kinesiophobia and pain catastrophizing can affect patient-reported outcomes following hip arthroplasty. Fourth, an a priori power analysis was not performed, so the analysis may be underpowered. Lastly, all of the surgical procedures were performed by a single fellowship-trained surgeon, with extensive experience in hip arthroplasty and the outcomes of this study may not be generalizable.

## Conclusions

There was a high prevalence of kinesiophobia among patients who underwent THA. Pain intensity directly correlated with the presence of kinesophobia whereas patients who had avascular necrosis and those who underwent physiotherapy were the most affected by kinesophobia. High pain intensity was associated in correlation with kinesiophobia and the result of this study will help the orthopedic surgeon in identifying who is most likely to suffer from high kinesiophobia. The results indicate that potential interventions regarding kinesiophobia should aim to decrease pain intensity. Further researches are needed in order to determine the exact prevalence rate of kinesiophobia in our region.
